# Methodology for the Early Detection of Damage Using CEEMDAN-Hilbert Spectral Analysis of Ultrasonic Wave Attenuation

**DOI:** 10.3390/ma18143294

**Published:** 2025-07-12

**Authors:** Ammar M. Shakir, Giovanni Cascante, Taher H. Ameen

**Affiliations:** Department of Civil and Environmental Engineering, University of Waterloo, 200 University Avenue W, Waterloo, ON N2L 3G1, Canada; amshakir@uwaterloo.ca (A.M.S.); taher.hama.ameen@uwaterloo.ca (T.H.A.)

**Keywords:** damage detection, HHT, CEEMDAN, HAS, attenuation, concrete

## Abstract

Current non-destructive testing (NDT) methods, such as those based on wave velocity measurements, lack the sensitivity necessary to detect early-stage damage in concrete structures. Similarly, common signal processing techniques often assume linearity and stationarity among the signal data. By analyzing wave attenuation measurements using advanced signal processing techniques, mainly Hilbert–Huang transform (HHT), this work aims to enhance the early detection of damage in concrete. This study presents a novel energy-based technique that integrates complete ensemble empirical mode decomposition with adaptive noise (CEEMDAN) and Hilbert spectrum analysis (HSA), to accurately capture nonlinear and nonstationary signal behaviors. Ultrasonic non-destructive testing was performed in this study on manufactured concrete specimens subjected to micro-damage characterized by internal microcracks smaller than 0.5 mm, induced through controlled freeze–thaw cycles. The recorded signals were decomposed from the time domain using CEEMDAN into frequency-ordered intrinsic mode functions (IMFs). A multi-criteria selection strategy, including damage index evaluation, was employed to identify the most effective IMFs while distinguishing true damage-induced energy loss from spurious nonlinear artifacts or noise. Localized damage was then analyzed in the frequency domain using HSA, achieving an up to 88% reduction in wave energy via Marginal Hilbert Spectrum analysis, compared to 68% using Fourier-based techniques, demonstrating a 20% improvement in sensitivity. The results indicate that the proposed technique enhances early damage detection through wave attenuation analysis and offers a superior ability to handle nonlinear, nonstationary signals. The Hilbert Spectrum provided a higher time-frequency resolution, enabling clearer identification of damage-related features. These findings highlight the potential of CEEMDAN-HSA as a practical, sensitive tool for early-stage microcrack detection in concrete.

## 1. Introduction

Early damage detection in concrete is crucial to ensuring the long-term durability of concrete structures. While advances in non-destructive testing (NDT) methods have made considerable progress in damage detection, the focus often remains on identifying existing damage rather than detecting it in its early stages. Previous studies have employed techniques such as infrared detection, electromagnetic waves, coda waves, concrete resistivity, and acoustic emission, among others, to detect structural damage in concrete [1,2,3]. However, early detection remains challenging, as many methods lack the sensitivity to detect minor defects or fail to account for the nonlinear, nonstationary nature of damage signals in concrete [4].

Current research on early damage detection primarily centers on ultrasonic NDT approaches, which are categorized into linear and nonlinear methods [5]. Linear methods, such as ultrasonic pulse velocity (UPV), pulse-echo, and impact-echo techniques, provide a straightforward approach to analyzing ultrasonic properties like wave velocity and attenuation [6]. In contrast, nonlinear methods, including higher harmonic and subharmonic generation, nonlinear wave modulation, and nonlinear resonance frequency shift, offer increased sensitivity to micro-cracks in lab-scale experiments [7,8,9]. Both approaches, however, are influenced by factors such as concrete’s material properties, defect characteristics, and signal processing limitations, often assuming linearity and stationarity in signal analysis [10].

In practice, concrete structures often exhibit nonlinear and nonstationary signal behaviors due to factors such as multiple reflections and interactions. However, conventional signal processing techniques, including the Fourier and Wavelet transforms, are limited under this assumption of stationarity and linearity [11,12]. While certain Fourier and Wavelet-based approaches address certain stationarity issues, they still struggle with nonlinearity and frequency resolution [13,14]. Recently, Hilbert–Huang Transform (HHT) has gained popularity for addressing such complex signal processing limitations. HHT is particularly effective for analyzing nonlinear and nonstationary signals, and has been demonstrated to precisely characterize damage in concrete by detecting changes in wave attenuation [15]. While HHT shows promise, it also faces challenges such as noise susceptibility and mode mixing, which are significant issues in Empirical Mode Decomposition (EMD) and Ensemble EMD (EEMD) [16].

Several studies support the potential of wave attenuation analysis and advanced time-frequency analysis to detect internal damage across various materials. For instance, Yim et al. [17] introduced an experimental technique for measuring wave attenuation from ultrasonic NDT signals. This work demonstrated that attenuation may offer more reliable information than wave velocity for evaluating damage in concrete. Similarly, Jiang et al. [18] investigated wave attenuation and frequency content using the UPV method, combined with Fourier and Wavelet transforms, to assess the compaction level and “dynamic” Young’s modulus of asphalt concrete specimens. Fartosy et al. [19] explored wave velocity and attenuation in thin fractures within homogeneous polymethylmethacrylate (PMMA) specimens using the UPV method. Their findings revealed a minimal decrease (less than 4%) in wave velocity; however, they noted a significant shift (up to 60%) in wave attenuation. Moreover, Nie et al. [20] validated the enhanced sensitivity of nonlinear ultrasonic methods compared to traditional velocity measurements in concrete specimens. The work conducted by Nie et al. [20] further demonstrated that nonlinear ultrasonic coefficients associated with signal amplitudes are more responsive to microcracks. Additionally, Wiciak et al. [21] proposed a wavelet synchrosqueezed transform (WSST)-based technique to increase the sensitivity of localized damage detection in cementitious materials, achieving an up to 36% improvement in attenuation analysis compared to conventional velocity-based approaches. These examples illustrate that wave attenuation can potentially serve as a tool for identifying the early stages of damage in concrete members [22].

In an advanced time-frequency analysis, Zhu and Law [23] employed HHT to examine the nonlinear damage characteristics of a reinforced concrete beam. They decomposed vibration data using EMD and computed the Hilbert–Huang Spectrum (HHS) for each Intrinsic Mode Function (IMF), effectively correlating variations in frequency and damping with crack behavior. Similarly, Antonio Jr. and Hirose [24,25] combined HHT with Discrete Wavelet Transform (DWT) and Synthetic Aperture Focusing Technique (SAFT) imaging to further improve defect detection in concrete. Musolino et al. [26] also found that HHT provided more reliable results than DWT when analyzing ultrasonic testing data on concrete and masonry walls. Bandara et al. [27] applied HHT for damage detection in timber utility poles; this study successfully demonstrated the effectiveness of HHT in identifying defects, emphasizing its potential for enhancing condition assessments. Additionally, Mousavi et al. [28] used complete ensemble empirical mode decomposition with adaptive noise (CEEMDAN) to conduct experimental evaluations of a CEEMDAN-Hilbert Transform–Artificial Neural Network (CEEMDAN-HT-ANN) model. This combined model merged data analysis with a machine learning approach to identify the presence, location, and severity of damage in a laboratory-model steel truss bridge. Consequently, there is an ongoing need for a reliable signal processing technique that can effectively handle nonstationary and nonlinear signals and surpass the decomposition issues to detect early-stage damage in concrete. To bridge this gap, it is important to clarify how HHT supports NDT applications. HHT addresses the key limitations of conventional methods, particularly for nonlinear, nonstationary ultrasonic signals. Unlike linear methods, HHT adaptively decomposes complex waveforms into interpretable components, enabling precise time-frequency analyses of damage-induced attenuation shifts. A comparison of signal processing techniques is provided in Table 1.

This study introduces a novel approach for enhancing early damage detection in concrete by combining two branches of Hilbert–Huang Transform: Complete Ensemble Empirical Mode Decomposition with Adaptive Noise (CEEMDAN) and Hilbert Spectral Analysis (HSA). The key innovations and contributions of this work include the following:

1. Enhanced Sensitivity to Early Damage: Unlike conventional methods, the proposed technique effectively handles nonlinear, nonstationary ultrasonic signals and resolves decomposition challenges (mode-mixing and noise-sensitivity), significantly improving early damage sensitivity.
2. Superior Frequency Resolution: By focusing on ultrasonic wave attenuation, the method achieves a higher frequency resolution, enabling more precise damage characterization in concrete specimens compared to full-structure analyses.
3. Energy-Preserving IMF Selection: A systematic selection of the most effective Intrinsic Mode Functions (IMFs) retains over 20% of the original signal’s energy, ensuring meaningful feature extraction while minimizing noise interference.
4. Time-Domain Damage Assessment: A modified energy-based damage index exclusively correlates positive IMF energy loss with severity, enabling robust time-domain assessment.
5. Experimental Validation and Robustness: The method has been validated on PMMA specimens with controlled cracks and on concrete under freeze–thaw cycles, demonstrating its robustness. A dedicated CEEMDAN repeatability analysis confirms consistency under noise, reinforcing reliability.

This framework provides a more accurate, noise-resistant, and practical solution for early damage detection, addressing critical gaps in ultrasonic-based structural health monitoring.

## 2. Methods

### 2.1. Hilbert–Huang Transform (HHT)

The HHT, proposed by Huang et al. [30], is a data analysis technique designed to extract information across the time and frequency domains, which is especially effective for nonlinear, nonstationary data. HHT operates in two stages: empirical mode decomposition and Hilbert spectral analysis [31].

#### 2.1.1. Complete Ensemble Empirical Mode Decomposition with Adaptive Noise (CEEMDAN)

**Figure 1:**
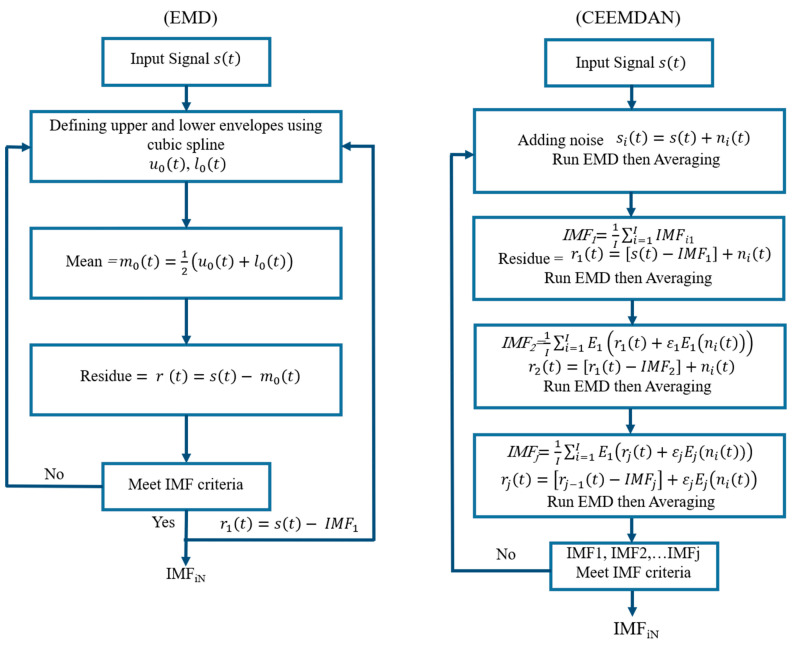
Flow charts of the “sifting process” of EMD and CEEMDAN for a given time series s(t): E_j (∙) is the operator that produces the j-th mode obtained by EMD, and (ε) is the coefficient that allows for the selection of the signal-to-noise ratio at each stage.

Empirical Mode Decomposition, introduced by Huang et al. [30], is a time series decomposition method that divides any signal into a finite set of intrinsic mode functions in the time domain [32]. IMFs represent narrow-band frequency components embedded in the original signal. For each IMF, the sum of the maxima and minima, as well as the number of zero-crossings, must differ by one. The resulting mean value of the upper and lower envelopes of the signal is nearly zero [30,33]. IMFs are thus narrow-banded and approximately symmetric relative to the time axis [34]. The EMD algorithm (see Figure 1) iteratively constructs IMFs by joining local extrema using cubic spline interpolation [30,35]. Each subsequent IMF captures progressively lower frequencies, with the final IMF representing the signal’s trend [36]. Mode mixing, a key challenge in EMD, occurs when different scales co-exist within a single IMF or when similar scales appear across IMFs, and can blur frequency distinctions. This issue is addressed by the Ensemble EMD technique, which integrates white Gaussian noise for scale separation [37]. However, EEMD may still include residual noise, potentially generating spurious modes [38]. The CEEMDAN procedure further refines EEMD by adaptively minimizing noise, resulting in cleaner signal decomposition. The sifting process of the CEEMDAN is illustrated in Figure 1 (right) [39].

The target signal, s(t), can be expressed in terms of the N modes as

(1) $$s\left(t\right)={\sum_{j=1}^{N}IMF}_{j}+r_{N}\left(t\right),$$

where N is the total number of IMFs, and r_N_(t) is the residue component. The final number of modes is determined only by the data and the stopping criterion [40,41].

#### 2.1.2. Hilbert Spectral Analysis (HSA)

**Figure 2:**
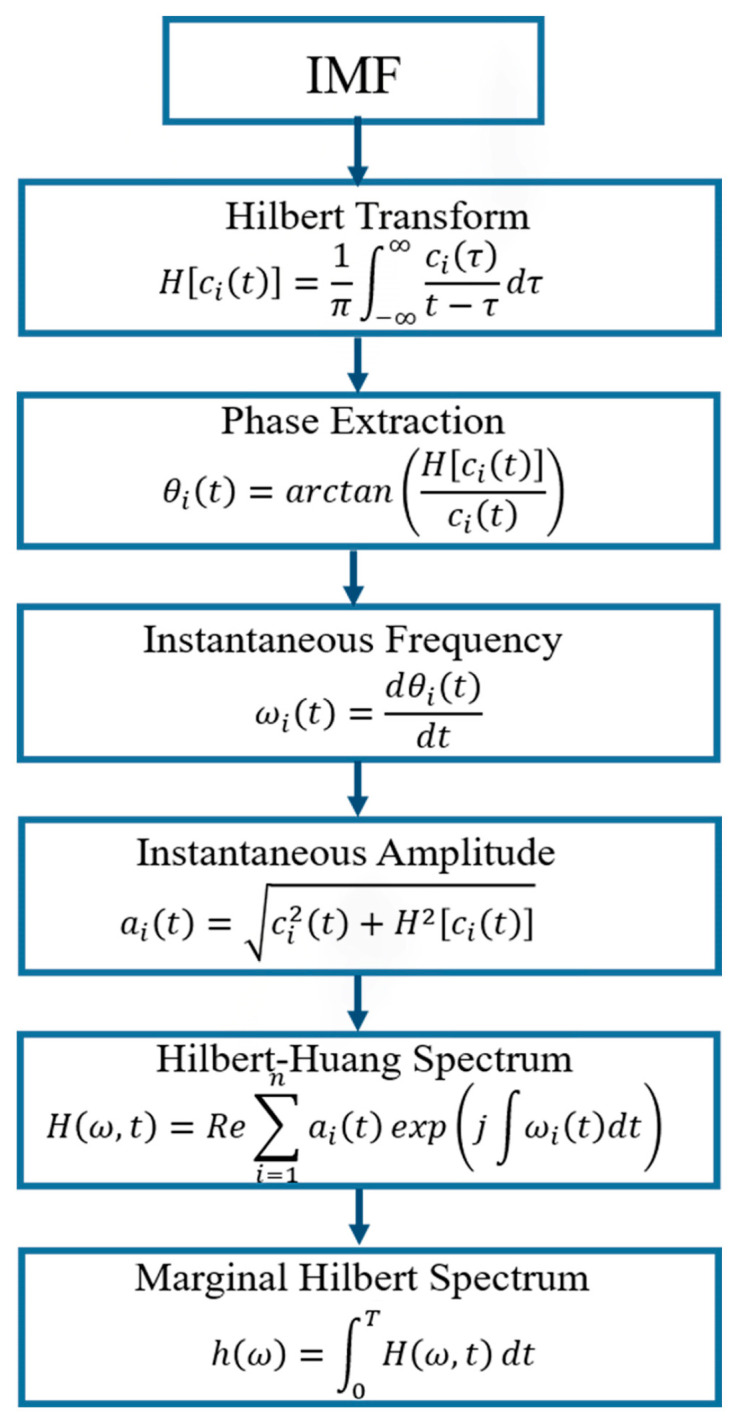
Flow chart of the Hilbert spectral analysis, where (α_i_(t)) is the IMF component and Re{.} denotes the real part of a complex quantity.

In the HSA, the instantaneous characteristic amplitudes and frequencies are obtained from IMFs as a time-frequency distribution of signal amplitude (or energy), which allows for the identification of localized features [36]. The instantaneous amplitude and angular frequency are determined by the amplitude and the derivative of each IMF’s unwrapped phase [42,43]. The main steps for amplitude and frequency extraction from an IMF are summarized in Figure 2 [30,42,44]. The local marginal spectrum, hi(ω), measures the total amplitude contribution from a given frequency (ω) and is used later in this study.

### 2.2. Selection of the Effective IMFs

The output of the CEEMDAN reveals several effective IMFs. These IMFs represent the real part of the signal and contain most of the signal information, as well as other ineffective IMFs that may represent the noise. The effective IMFs typically represent the IMFs that are most affected by the damage [45]. The Pearson correlation coefficient (PC_C_), percent fit, and energy comparison were used in this study to evaluate the effectiveness of the produced IMFs. Pearson correlation was selected because it quantifies the linear similarity between each IMF and the original signal, helping to distinguish signal-bearing components from noise-dominated ones. The PCc function measures the strength and direction of a linear relationship between two signals; for this study, PCc is used to compare the original decomposed signal and its IMFs signals. Ineffective IMFs will have very small (or close to zero) PCc values compared to the original signal, allowing for quick determination of the effective IMFs [46]. The Pearson correlation coefficient has been widely used in signal processing and other fields [47,48]. The PCc function in terms of the covariance of the two random variables, A and B, can be defined as follows:

(2) $${PC}_{c}=\frac{cov\left(A,B\right)}{\sigma_{A}\sigma_{B}},$$

where σ_A_ and σ_B_ are the standard deviations of A and B, respectively.

The normalized root mean square error value (NRMSE) is also applied to examine the importance of the IMFs to produce the percent fit (Fit%) value as per Equations (3) and (4). This metric facilitates comparison between datasets or models with different scales. It is often expressed as a percentage, where lower values indicate less residual variance and better agreement between measured and calculated values.

(3) $$NRMSE=\sqrt{\frac{\frac{1}{N-1}\sum_{i=1}^{N}{\left(x_{i}-{IMF}_{i}\right)}^{2}}{\sum_{i=1}^{k}\frac{{\left(x_{i}\right)}^{2}}{N}}},$$

where N is the number of samples, and x_i_ is the original signal.

(4) $$Fit\%=\left(1-NRMSE\right)\times 100,$$

Each IMF’s energy, based on Equation (5) proposed by Cheraghi et al. [49] and adopted by Li et al. [50], is calculated to plot the energy curve of the IMFs, reflecting which IMF is more important and effective than the other IMFs.

(5) $$E_{i}=\int_{0}^{t0}{\left({IMF}_{i}\left(t\right)\right)}^{2}dt,$$

The energy of each IMF is calculated as the total squared amplitude of that mode over time, effectively quantifying the contribution of its associated frequency band to the overall signal. In ultrasonic non-destructive testing, the presence of damage alters wave propagation and often leads to localized energy attenuation or redistribution. As a result, analyzing the energy content of IMFs enables the detection of material degradation.

### 2.3. Damage Index (DI)

The Damage Index (DI) procedure helps study the effect of damage caused by loading on each IMF. Moreover, it also distinguishes various damage sizes [14]. Based on Cheraghi et al. [51], the decomposition results of the vibration signals traveling through the structure can be used to identify the damage in that structure. The first IMF energy (E) for the recorded signals is used; the index is then defined as the percentage difference in the structure’s energies between intact and damaged states. The damage index can be calculated as follows:

(6) $$DI=\frac{E_{Intact}-E_{Damaged}}{E_{Intact}}\times 100,$$

Cheraghi et al. [49,52] concluded that this method works well for cases where the first IMF represents the dominant vibrating frequency and suggests a band-pass filter for filtering data to keep the first frequency. Rezaei and Taheri [53] found that the first IMF does not always represent the dominant frequency, and they suggest that more frequency components should be included in the passband filter. In general, high DI values identify the existence of damage.

While the above formulation provides a reliable damage index under controlled conditions, its application in real-world structures is limited by the requirement for a reference intact signal. This requirement is difficult to meet in aging or in-service concrete structures, where baseline data may be unavailable or unreliable. Environmental and operational variations can further distort comparisons with historical data. In such cases, surrogate baselines from structurally similar elements or statistical modeling approaches may be used, although they introduce uncertainty. Emerging strategies, such as machine learning models trained to learn healthy signal characteristics or baseline-free damage detection frameworks, may offer promising alternatives, albeit with a trade-off in terms of their diagnostic specificity. Therefore, future work should explore hybrid approaches that adapt DI methodologies for practical field use.

## 3. Experimental Procedure

### 3.1. Methodology

This study introduces a novel signal processing technique to enhance the early detection of damage utilizing the HHT method with CEEMDAN. Firstly, this technique was validated using synthetic data. The technique was then applied to measurements from a homogeneous medium featuring a single crack, specifically using polymethylmethacrylate specimens. The method’s ability to assess early damage was demonstrated through measurements of concrete specimens that underwent microfractures due to freeze–thaw cycles. Four prismatic concrete specimens (10 cm × 10 cm × 40 cm) were cast using a Portland cement mix with a water-to-cement ratio of 0.40, aggregates (12.5 mm max), and air-entraining admixture (AEA) dosed at 0%, 0.1%, 0.125%, and 0.25% by cement mass, following ACI 211.1R-91. All specimens were moist-cured at 20 °C for 28 days. Prior to freeze–thaw cycling, they were submerged in water for four days to ensure saturation. The freeze–thaw exposure lasted eight weeks, with daily temperature cycles ranging gradually from −25 °C to +25 °C (±2 °C), simulating progressive environmental deterioration. This exposure protocol was based on ASTM C666 Procedure A [54], which uses 300 cycles as a benchmark for evaluating concrete’s resistance to freeze–thaw damage. These cycles are widely recognized to induce early-stage microcracking, particularly in the cement matrix and at the aggregate interface [55]. The resulting microcracks are typically not visible to the naked eye and are expected to be smaller than 0.5 mm in width, consistent with previously reported observations [56]. Finally, the robustness of the CEEMDAN approach was evaluated statistically.

### 3.2. Experimental Setup

**Figure 3:**
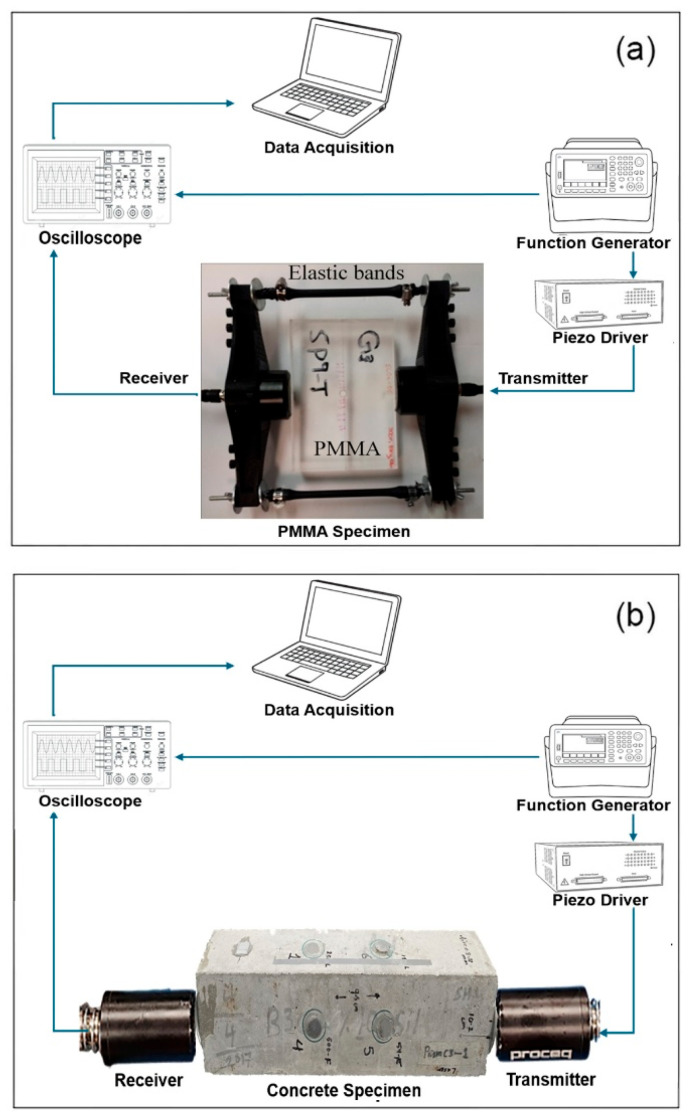
The schematic testing system of ultrasonic pulse velocity instrumentation setup for PMMA (**a**) and concrete (**b**) specimens.

The experimental setup for data acquisition includes a function generator, an oscilloscope, and a data acquisition system. Transmitter and receiver transducers were attached to the sample at specified locations using fastening attachments. For the PMMA sample, transducers with a nominal frequency of 54 kHz and a diameter of 50 mm were held in place by an elastic cord within a plastic holder (Figure 3a). Interfacial coupling grease was applied to minimize signal loss due to air voids [57]. To reduce the random noise during testing, signal averaging was employed, with 16 waveforms per measurement for PMMA and 32 waveforms for concrete specimens. A standard deviation of less than 0.01% was regarded as acceptable. Concrete specimens were prepared and exposed to freeze–thaw cycling, as described in the Methodology section, with four prismatic samples tested under consistent environmental and ultrasonic conditions. The same setup and transducers were utilized for testing the concrete specimens, ensuring consistent coupling with Sonotech Ultragel II, Figure 3b. The coupling process, transducer alignment, and specimen positioning were standardized, and each test was repeated multiple times at the same location for both intact and damaged samples to account for variations.

## 4. Results and Discussion

### 4.1. Validation of Decomposition Completeness

**Figure 4:**
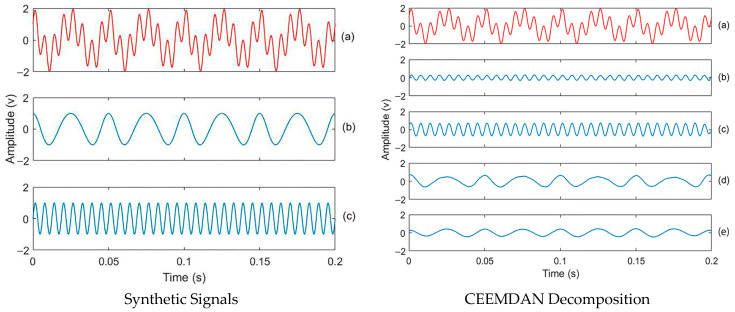
Signal decomposition validation: (**left**) synthetic signals. (**a**) Signal x(t), (**b**) Signal x_1,_ and (**c**) Signal x_2_; (**right**) CEEMDAN decomposition results of signal x(t). (**a**) original signal; (**b**–**e**) represent IMF3–IMF6, respectively.

CEEMDAN’s ability to perform a complete decomposition was validated using a synthetic signal composed of multiple frequency components: a low-frequency component, signal x_1_ (40 Hz, 20 Hz modulation), and a high-frequency component signal x_2_ (160 Hz), Figure 4 (left). Using a sampling rate of 2560 Hz, the CEEMDAN algorithm produced nine IMFs, sorted by frequency. Summing these IMFs and the final residue accurately reconstructed the synthetic signal. The reconstructed signal demonstrated minimal reconstruction error (less than 10^−14^), indicating complete decomposition. This confirms the correctness of CEEMDAN decomposition, providing evidence that the extracted IMFs represent true physical components of the signal and are not artifacts of the algorithm. Effective IMFs (IMF3–IMF6) were identified for reconstructing high- and low-frequency components with minimal error, as shown in Figure 4 (right).

### 4.2. Testing of HHT on PMMA Samples

**Figure 5:**
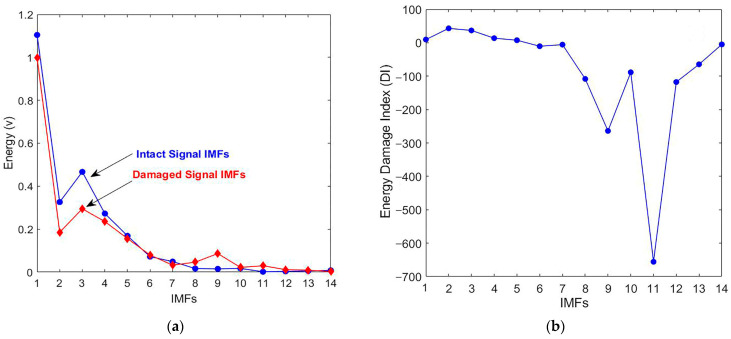
(**a**) Energy comparison of the CEEMDAN-produced IMFs of the PMMA sample. (**b**) Energy-based damage index curve of the CEEMDAN-produced IMFs of the PMMA specimen.

To examine the feasibility of the damage detection technique, experiments were carried out on transparent and homogenous PMMA specimens, which are commonly utilized in fracture evaluation studies. The first test was conducted on an intact sample, while subsequent tests involved a damaged specimen with a single crack. CEEMDAN decomposed the acquired signals into fourteen IMFs; the effective IMFs exhibited high similarity and correlated with the decomposed signal, which allowed for the reconstruction of the original signal with minimal error. The energy for each IMF was calculated, and the energy curve is outlined in Figure 5a. The damage index curve was plotted to determine the damage state (Figure 5b). The results illustrate how effective IMFs are affected by the presence of damage.

Negative DI values in IMFs 6–14 indicate increased energy in the damaged signal, primarily resulting from nonlinear wave–damage interactions (e.g., crack-induced harmonics), energy redistribution to lower-frequency components, or noise amplification. While these phenomena are physically meaningful, our statistical validation confirms that they lack consistency as damage indicators, showing no predictable relationship with the progression of severity. This approach aligns with (1) Cheraghi et al.’s [49] emphasis on dominant-frequency IMFs that yield stable positive DI values correlating reliably with structural degradation, and (2) Rezaei and Taheri’s [53] observation that non-dominant IMFs require careful filtering to avoid spurious energy shifts—a finding supported by our robustness analysis. The PMMA specimen signals’ decomposition results were examined in the time-frequency and frequency domains to check Hilbert’s spectral analysis for damage identification. From the HHT, the instantaneous characteristics (instantaneous amplitude and instantaneous frequency) are considered unique results. The instantaneous amplitude is the magnitude envelope of the analytic signal given by HHT, while the instantaneous frequency represents the signal’s frequency behavior over time. This behavior is highly localized in the time-frequency domain and reveals important local characteristics of the nonstationary signal. HSA defines the local attributes of a nonstationary signal, the instantaneous frequency, and the instantaneous amplitude of the examined IMFs. Consequently, the resulting Hilbert spectrum provides sharp identifications of embedded structures at specific frequencies.

Using HSA, the resulting IMFs can be examined to define the local instantaneous characteristics. The calculated energy damage index values for the instantaneous characteristic of the intact and damaged PMMA specimens’ signals and their most effective IMFs strongly support the HHT’s function as a signal processing tool for damage identification. The Marginal Hilbert Spectrum (MHS), which reflects the total amplitude (or energy) contribution at each frequency, captured changes in frequency components associated with damage. The results listed in Table 2 clearly showed that the technique could detect an energy reduction of up to 88% of the original energy due to damage.

The Damage Index (DI) values in Table 2 were calculated using Equation (6), based on the relative energy loss between intact and damaged signals for each IMF. Each IMF corresponds to a distinct frequency band, determined through Hilbert spectral analysis. Damage induces energy attenuation in specific frequency ranges, which is reflected in the higher DI values. The IMFs with the highest DI values (e.g., those between 30 and 70 kHz) align with the transducer’s center frequency and reveal the frequency bands most sensitive to material degradation. Although the DI values from FFT and MHS are numerically close, the MHS provides a higher time-frequency resolution. This enables the clearer identification of transient, damage-related features and amplitude fluctuations within specific frequency bands, details that are often averaged out in the FFT’s global spectral analysis. Such resolution is particularly beneficial for diagnosing early-stage micro-damage.

### 4.3. Application of HHT in Concrete Samples

**Figure 6:**
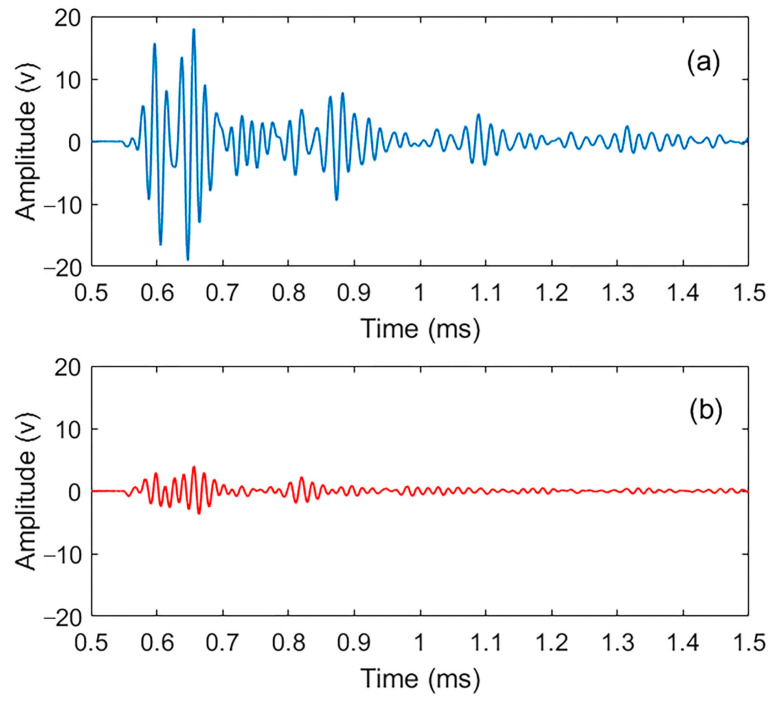
Ultrasonic signals for the concrete sample in both (**a**) intact and (**b**) damaged states.

To identify damage, concrete specimens were tested using a non-destructive ultrasonic test in their intact state and after generating micro-damage through 300 cycles of freeze and thaw. All specimens showed consistent responses under identical test conditions. The 0.125% AEA specimen was chosen for detailed presentation as it best represented the median values of both energy attenuation and frequency response. The corresponding vibration signals are shown in Figure 6, with a time window between 0.5 ms and 1.5 ms chosen to represent the signal’s vibration over a relevant period.

The CEEMDAN algorithm was applied to decompose the signals into their main frequency components, identifying fourteen intrinsic mode functions (IMFs) for the intact and damaged specimen signals. These IMFs represent various frequency components and residues. It is important to note that no filtering was applied to the signals, as removing noise could also eliminate important frequencies containing valuable information. To ensure CEEMDAN’s robustness, 100 decomposition trials were conducted for each signal (intact and damaged). This high repeatability across realizations emphasizes the applicability of CEEMDAN in identifying damage-sensitive modes, even in the presence of signal variability. Across all trials, CEEMDAN demonstrated a consistent and repeatable performance in generating dominant intrinsic mode functions (IMFs), particularly IMFs 2–5, which exhibited a stable energy distribution, high correlation with the original signal, and low statistical variation in both kurtosis and spectral entropy metrics. This demonstrates the method’s robustness, reliability, and suitability for reliable early-stage damage detection in concrete.

**Figure 7:**
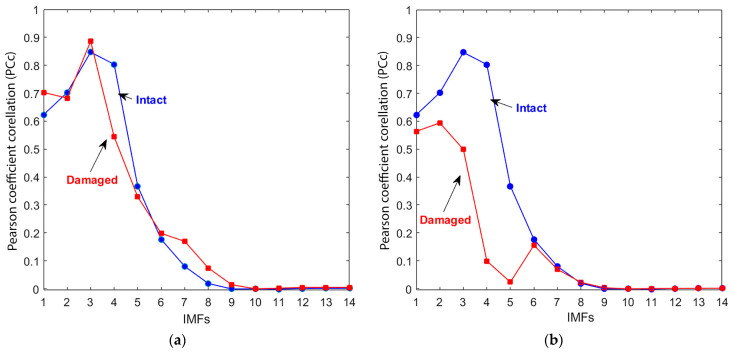
The correlation coefficient values for the IMFs of both intact and damaged concrete sample signals. (**a**) Each IMFs correlated to its original signals; (**b**) both IMFs correlated to intact signals.

The correlation coefficients for the CEEMDAN output were calculated by comparing the IMFs to their original signals (Figure 7a). IMFs 1–6 showed the highest Pearson correlation coefficients (PCc > 0.2), indicating they had the most significant similarity to the original signal. In contrast, IMFs 7–14 had correlation values close to zero, suggesting they are less relevant and can be disregarded in damage analysis. The Pearson correlation coefficient was used to evaluate the similarity between each IMF and the original signal. Higher PCc values indicate that the IMF retains meaningful signal features, while lower values suggest the dominance of noise. A PCc > 0.2 was empirically chosen based on our dataset’s distribution to identify IMFs that retain significant structural similarity to the original signal. Although relatively low in general statistical terms, this threshold is appropriate for decomposed ultrasonic signals, particularly under noisy conditions. Similar correlation-based approaches for IMF selection have proven effective in other signal processing applications [58].

To investigate the presence of damage, the IMFs of both signals were compared to the intact specimen signal. Figure 7b illustrates how the effective IMFs of the damaged specimen signal show a distinct contrast compared to the intact signal’s IMFs, providing insight into the damaged state of the concrete specimen through the observed shifts in the curves.

**Figure 8:**
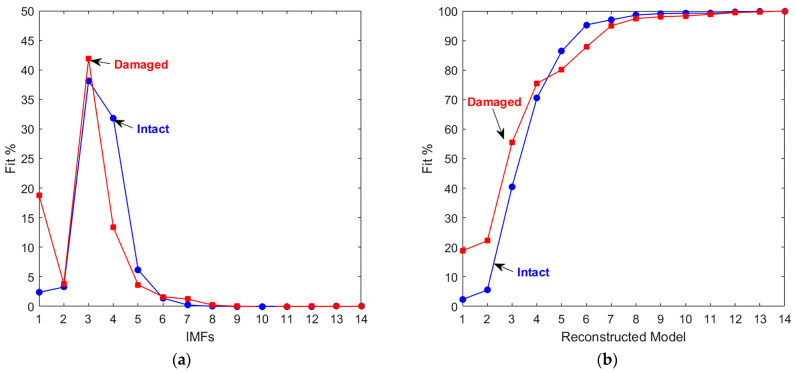
Comparison of NRMSE percent fit values of intact and damaged sample IMFs. (**a**) IMFs and (**b**) IMFs reconstructed models.

Equations (3) and (4) were applied to calculate the normalized root mean square error (NRMSE) and the percent fit of each IMF. The results indicate that IMFs 1–6 provide the highest percentage of fit, meaning they best represent the original signal (Figure 8a). To define the damage signature in a damaged concrete specimen by using the NRMSE procedure, reconstructed models were produced by cumulatively adding the IMFs and modifying Equation (3) by replacing the IMF_i_ with a reconstructed model, M_k_, that can be defined as follows:

(7) $$M_{k}=\sum_{i=1}^{k}{IMF}_{i},$$

where k = 1, 2, ……, the last IMF.

The percent fit for the reconstructed models was calculated according to Equation (4), and the damage signature was revealed by the shifts in the NRMSE curves when comparing the models for both the intact and damaged signals (Figure 8b).

The energy of each IMF for both intact and damaged signals was calculated using Equation (5), and the resulting energy curves were plotted to examine the effectiveness of the IMFs.

**Figure 9:**
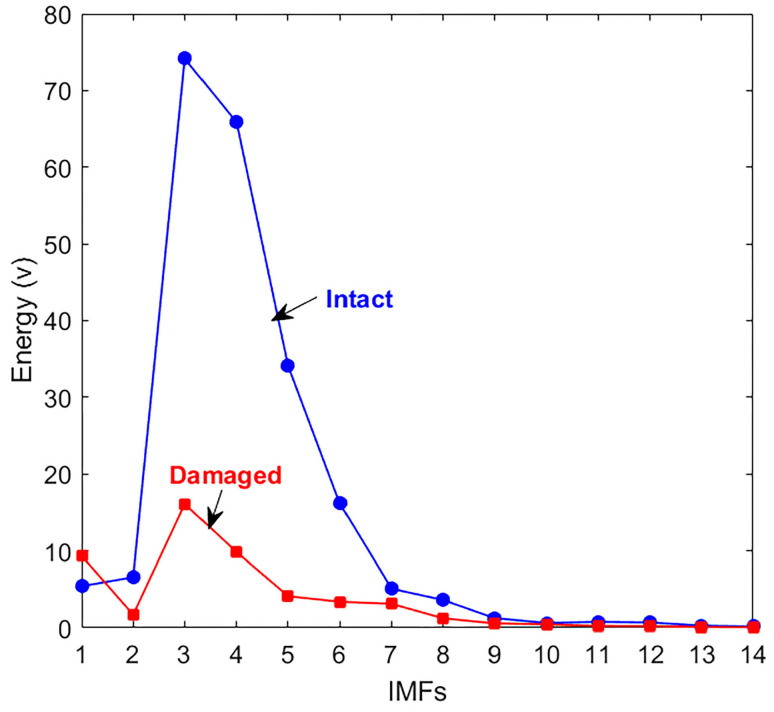
The energy comparison for the IMFs of intact and damaged concrete sample signals.

**Figure 10:**
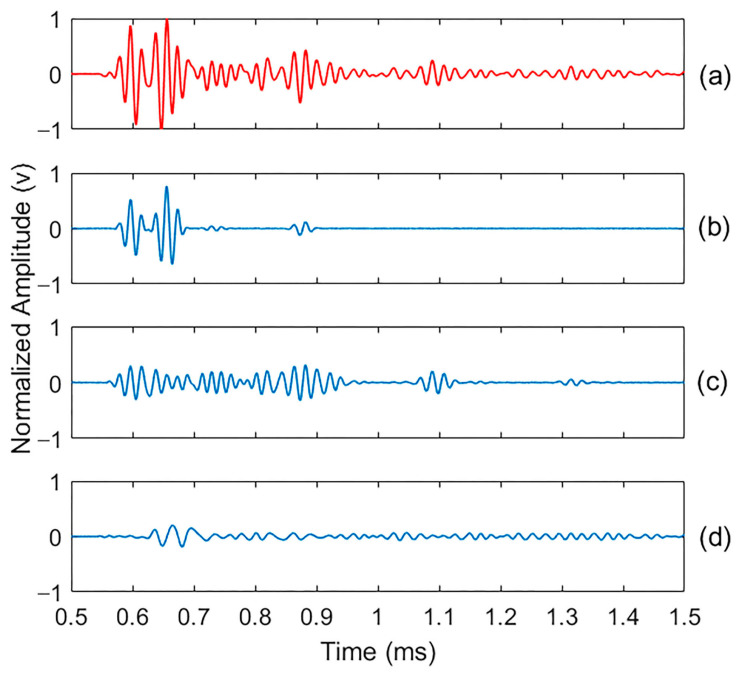
Normalized intact sample signal and the three most important IMFs of effective CEEMDAN decomposition results: (**a**) original signal; (**b**), (**c**,**d**) are IMF3, IMF4, and IMF5, respectively; the normalized factor is 18 (v).

**Figure 11:**
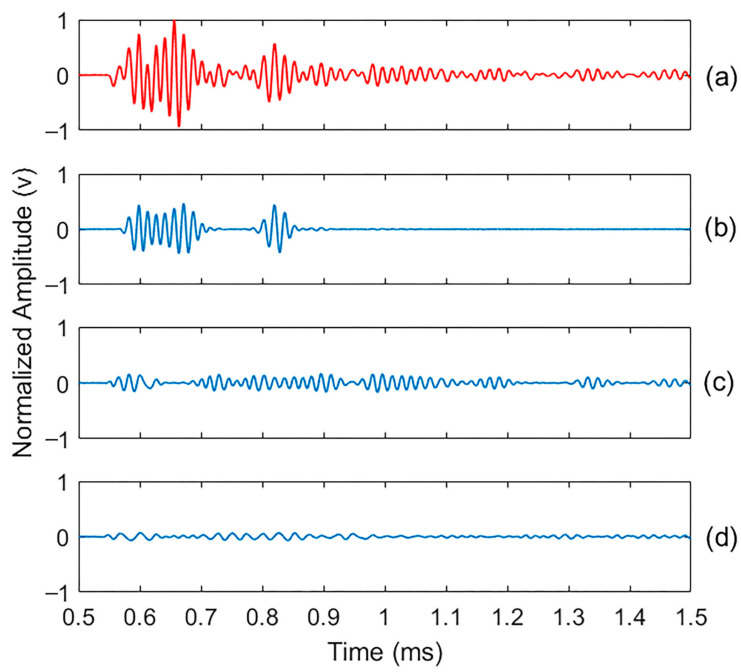
Normalized damaged sample signal and the three most important IMFs of effective CEEMDAN decomposition results: (**a**) original signal; (**b**), (**c**,**d**) are IMF3, IMF4, and IMF5, respectively; the normalized factor is 4 (v).

As shown in Figure 9, some IMFs exhibit significantly higher energy values than others. To define the effective IMFs, only those with more than 20% of the original decomposed signal’s energy were selected. A 20% energy threshold was employed to select effective IMFs for damage analysis. This criterion was based on empirical observations that IMFs surpassing this threshold consistently retained key waveform features and exhibited high correlation with the original signal, while those below the threshold were predominantly noise-dominated and contributed minimally to the signal’s energy. This energy-based filtering aligns with prior studies that use similar thresholds to differentiate meaningful modes from background noise in empirical decomposition analyses [59]. Consequently, IMFs 3–5, for both the intact and damaged signals, were determined to be the most effective IMFs. These components demonstrated a stronger correlation and higher similarity to the original signals and were able to reconstruct the signals with less than 10% error, as shown in Figure 10 and Figure 11.

#### 4.3.1. Damage Identification in Time Domain

**Figure 12:**
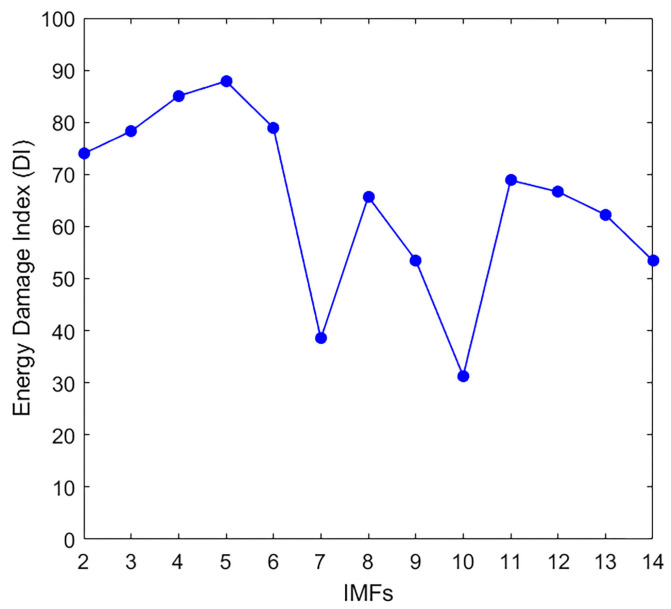
Energy damage index values of the produced IMFs are calculated in the time domain.

In the time domain, the CEEMDAN output reveals that the first IMF does not represent the dominant frequency component. Instead, the other IMFs contribute a range of frequencies with high amplitude levels; this was assessed by calculating the DI for the concrete specimens without applying any filters. As shown in Figure 12, the damage index values indicate that IMFs 3–5 are most affected by the damage, while other IMFs (e.g., 1–2 and 6–14) show negative or smaller and inconsistent responses. Notably, IMF1 has a negative DI value and was therefore excluded from further analysis.

The DI curve obtained from this analysis helps assess the damage state by showing the response of high- and low-frequency components in the vibration signal under loading. Table 3 lists the DI values for the recorded signals and their most affected IMFs in the time domain and significant time ranges.

#### 4.3.2. Damage Identification in Time–Frequency Domain

The recorded signals were analyzed using the HSA method to study the influence of the damage on the propagation of various frequency components over time. As a result, the instantaneous frequency–time variation and distribution in the time–frequency representation of IMF amplitude were calculated and are presented as an energy–frequency–time distribution Hilbert spectrum.

**Figure 13:**
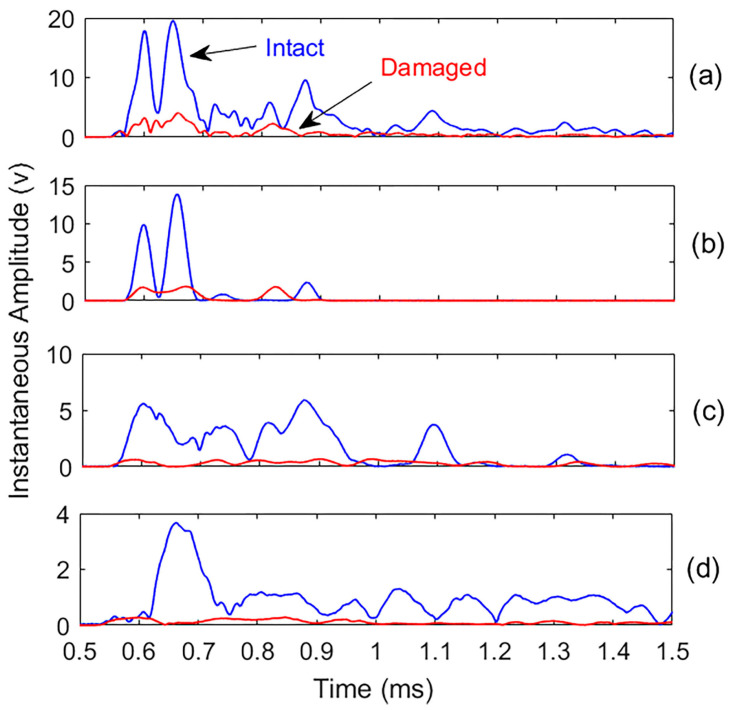
Comparison of the instantaneous amplitudes for intact (blue) and damaged (red) sample signals and their most effective IMFs: (**a**) original signals; (**b**), (**c**,**d**) are IMF3, IMF4, and IMF5, respectively.

**Figure 14:**
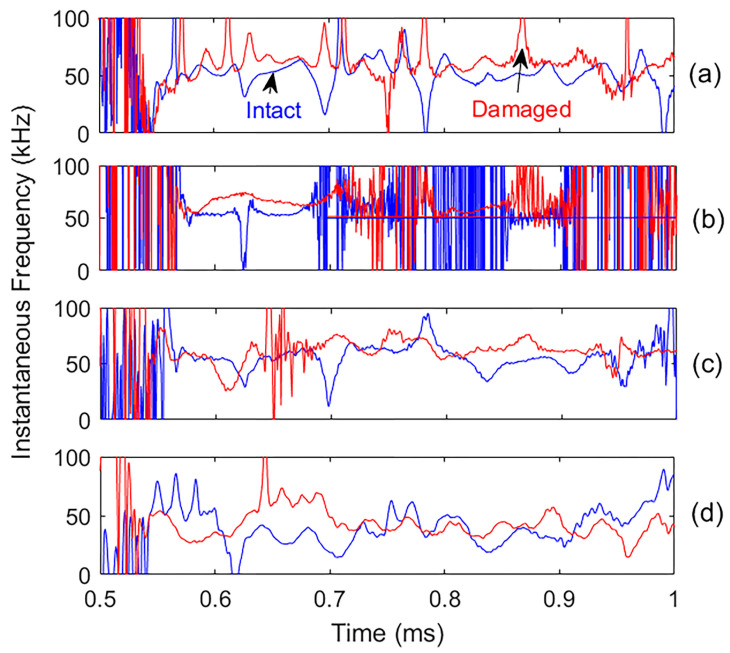
Comparison of the instantaneous frequencies for intact (blue) and damaged (red) sample signals and their most effective IMFs: (**a**) original signals; (**b**), (**c**,**d**) are IMF3, IMF4, and IMF5, respectively.

Instantaneous characteristics, including amplitude and frequency, were extracted from the recorded signals and their most effective IMFs for comparison. This analysis reveals a marked decrease in instantaneous amplitude due to the presence of damage, as shown in Figure 13. In contrast, the instantaneous frequencies display a slight increase and greater fluctuations in the damaged specimen (Figure 14), possibly due to increased damping in the damaged concrete compared to the intact one. The clear reduction in instantaneous amplitude provides a more reliable indicator of the presence of damage than changes in instantaneous frequency. The fluctuations in instantaneous frequency are primarily due to scattering and nonlinear wave interactions caused by internal cracking. While residual noise may also contribute, the increase in frequency variability in the damaged specimen reflects meaningful structural changes. However, in agreement with the broader analysis, an instantaneous amplitude reduction remains the more reliable damage indicator, while frequency fluctuation serves as a secondary, supportive metric.

Damage index values of the extracted instantaneous amplitude were calculated for each component’s effective time using Equation (6). Table 4 presents the calculated energy damage index values in the time–frequency domain.

#### 4.3.3. Damage Identification in the Frequency Domain

**Figure 15:**
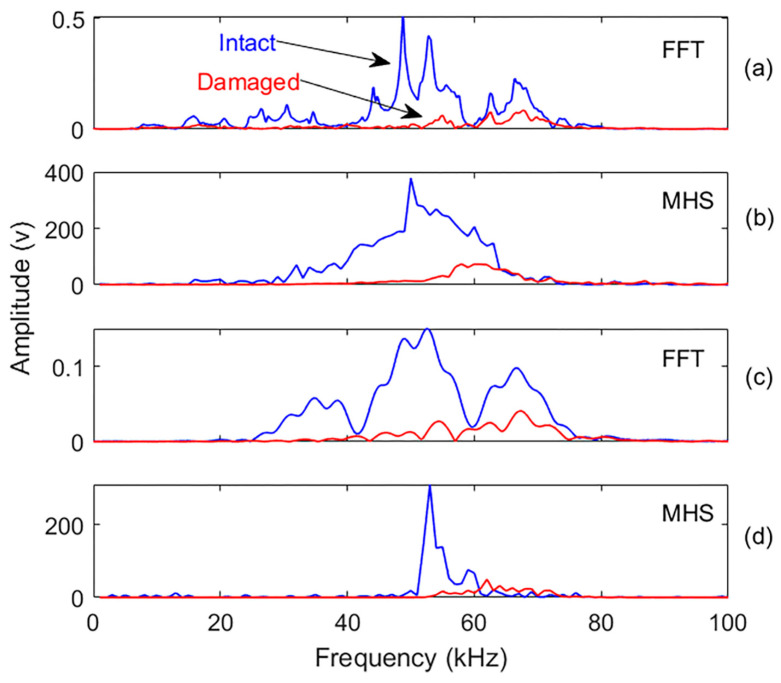
FFT spectra and MHS of both intact (blue) and damaged (red) sample signals and one of their most effective IMFs: (**a**) FFT of the original signals; (**b**) MHS of the original signals; (**c**,**d**) are the FFT and MHS of the IMF3, respectively.

The recorded signals and their most significant IMFs in the frequency domain were analyzed to further investigate amplitude reduction as a damage indicator. The MHS reflects the total energy contribution at each frequency and captures changes in frequency components associated with damage. A more traditional Fourier analysis assumes a stationary signal represented by persistent sine or cosine waves and can misrepresent the characteristics of nonstationary data, which leads to differing frequency components in HHT [60]. As such, the FFT spectrum is also included here for comparison with the MHS results. Figure 15 compares the FFT and MHS spectra for the recorded signals and their effective IMF 3.

**Figure 16:**
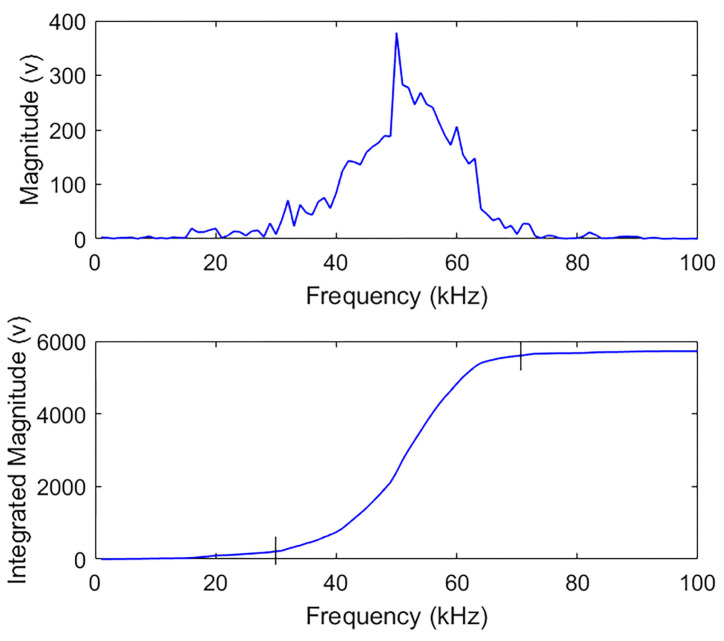
Determination of the effective frequency range on the MHS of the intact sample signal.

Figure 15 reveals a significant drop in spectrum magnitude for the damaged specimen’s signals and their IMFs, indicating the presence of damage. While both FFT and MHS detect frequency components, MHS achieves higher resolution by capturing time-dependent frequency variations rather than amplitude changes. To define the most effective frequency ranges, we performed a mathematical integration of the magnitude spectrum and plotted the cumulative magnitude versus frequency. The curvature changes observed in the integrated curve (see Figure 16) reveal the frequency interval most impacted by damage, identified here as approximately 30–70 kHz. This frequency band aligns with the 54 kHz center frequency and ±20 kHz effective bandwidth of the ultrasonic transducers used and is consistent with known attenuation behavior in PMMA and concrete. This range corresponds to the dominant energy band in both intact and damaged signals and was used to guide the selection of effective IMFs for further analysis. This frequency range was subsequently used to define the interval for damage index calculation, as described below.

A new damage index, reflecting the magnitude reduction, was then calculated for each signal’s effective frequency interval by comparing the area under the intact and damaged spectra. The maximum amplitude reduction was observed within the 30–70 kHz range, centered around the transducer frequency. Table 5 compares the damage index values for the recorded signals and their most effective IMFs within this effective frequency range.

The results demonstrate that damage notably alters signal characteristics, with amplitude loss exceeding 85% of its original energy. This significant reduction serves as strong evidence of damage to the concrete specimen. The results also demonstrate that MHS has about 20% more sensitivity than FFT.

### 4.4. Evaluation of Decomposition Robustness

A comprehensive statistical evaluation was performed to validate the robustness and reliability of CEEMDAN in extracting stable IMFs. This analysis consisted of two primary phases:

1. Assessing feature variability across multiple decomposition trials;
2. Evaluating consistency under increasing noise perturbations, focusing on CEEMDAN’s noise-assisted characteristics.

To facilitate comparative analysis, 100 independent decomposition trials were performed for each signal condition (intact and damaged) using CEEMDAN and traditional EMD. In CEEMDAN, each trial employed a distinct realization of white Gaussian noise. The first ten IMFs, excluding the residues, were extracted and analyzed. Two key statistical features were evaluated: Kurtosis and spectral entropy. Kurtosis, as the fourth-order moment, measures signal impulsiveness, often linked to localized structural damage. It is defined as follows:

(8) $$Kurt\left(x\right)=\frac{1}{N}\sum_{i=1}^{N}{\left(\frac{x_{i}-\mu }{\sigma }\right)}^{4},$$

where x_i_ is the data sample, μ is the mean, σ is the standard deviation, and N is the number of samples.

Secondly, spectral entropy, which is derived from the normalized power spectral density (PSD), quantifies spectral complexity. Higher entropy suggests more randomness; lower values indicate structural regularity:

(9) $$H=-\sum_{i=1}^{N}p_{i}{log}_{2}{\left(p_{i}\right)}^{4},$$

where p_i_ are the normalized PSD components.

For each feature, the standard deviation and coefficient of variation (CV) were calculated across trials to quantify variability. The CV is a normalized measure of dispersion, defined as follows:

(10) $$CV=\frac{\sigma }{\mu }$$

where σ is the standard deviation and μ is the mean.

Although CEEMDAN exhibited slightly higher raw standard deviations than EMD, it consistently yielded lower CV values, particularly for IMFs 3 to 5. These modes showed minimal variability and high feature stability, reinforcing their identification as the most informative components. While the initial selection of IMFs was based on energy content, the inclusion of kurtosis and spectral entropy further validated the reliability of IMFs 3 to 5.

**Figure 17:**
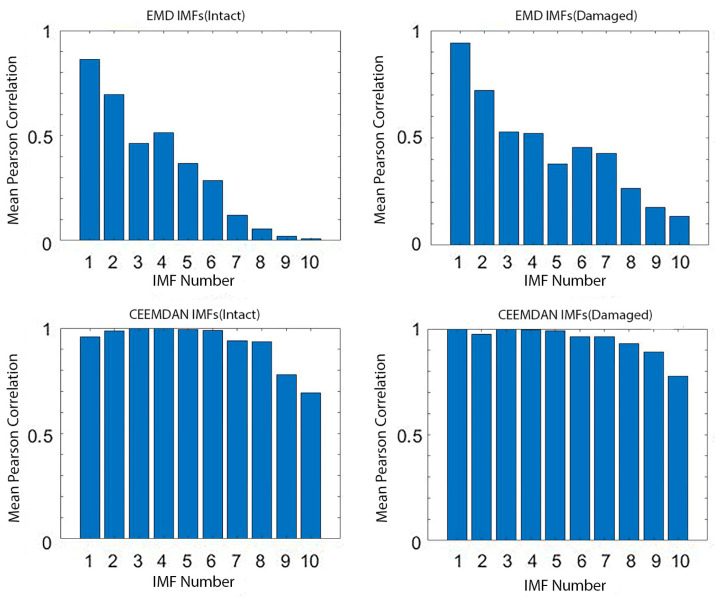
Mean Pearson correlation of EMD and CEEMDAN IMFs across 100 decomposition trials (intact and damaged signals).

To further quantify decomposition consistency, the Pearson Correlation Coefficient (PCc), as per Equation (2), was calculated for each IMF across all 100 trials (Figure 17). This metric captures the linear relationship between decompositions of the same IMF across trials.

The results indicated significantly higher average PCc values for CEEMDAN, with IMFs 2 to 5 consistently exceeding a correlation of 0.8, suggesting a superior level of repeatability. In contrast, IMF1 and IMFs 8 to 10 showed a lower correlation, likely due to noise sensitivity.

**Figure 18:**
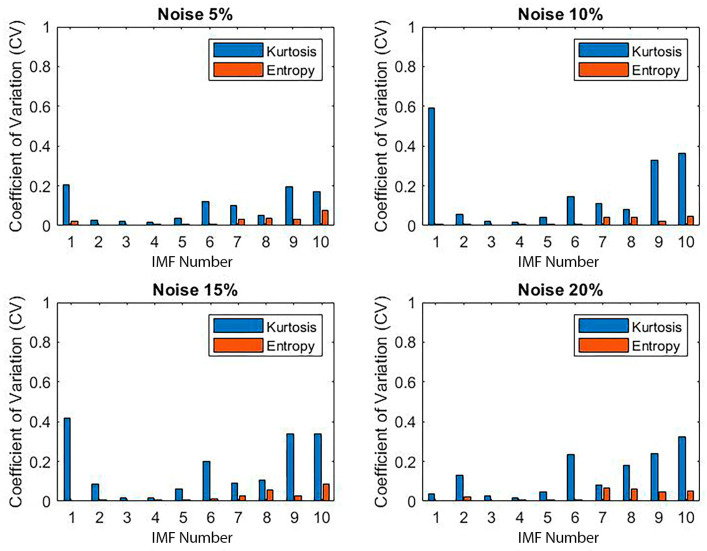
Coefficient of variation across 100 decomposition trials with varying noise levels (intact signal).

**Figure 19:**
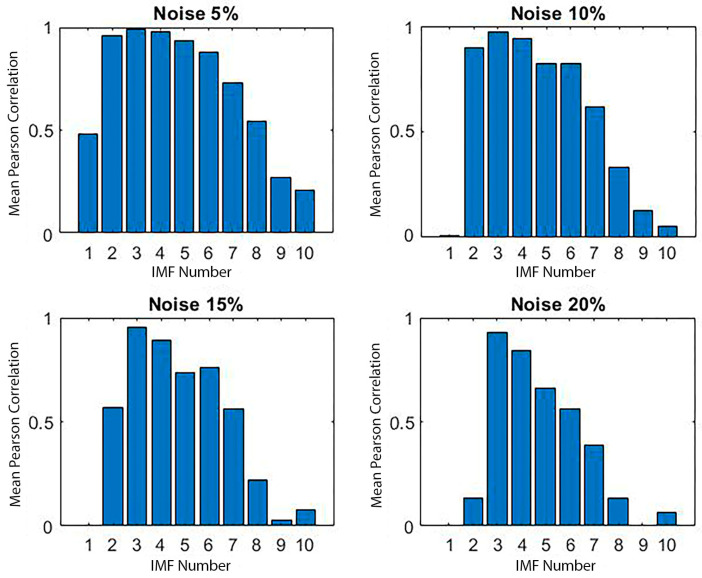
Mean Pearson correlation for each IMF across 100 trials at varying noise levels (damaged signal).

To explore decomposition stability under perturbation, we introduced controlled noise levels (5%, 10%, 15%, and 20%) to the signals and repeated the 100-trial decomposition process at each level. Despite the increase in noise intensity, IMFs 3 to 5 maintained a low CV and high correlation, demonstrating resilience (Figure 18 and Figure 19). Other IMFs, particularly IMF1 and higher-order modes, exhibited marked degradation, confirming prior observations.

This two-phase analysis confirms that CEEMDAN offers more robust and consistent IMF extraction than EMD, particularly under noisy conditions. Its inherent noise-assisted framework enhances resistance to variability without the need for external denoising. IMFs 3–5 consistently demonstrated optimal performance in terms of stability, correlation, and sensitivity to damage, making them highly suitable for structural health monitoring and early damage detection.

## 5. Conclusions

This study introduces a novel energy-based approach for early damage detection in concrete through integrating CEEMDAN with HAS through the Hilbert–Huang Transform framework. Unlike conventional Fourier and Wavelet-based methods, this technique effectively processes nonlinear, nonstationary ultrasonic signals while mitigating decomposition-related challenges, significantly improving sensitivity to early-stage damage.

The proposed method enhances frequency resolution and enables precise damage assessment at the specimen level by focusing on ultrasonic wave attenuation. CEEMDAN decomposes ultrasonic signals into frequency-ordered IMFs, addressing the mode-mixing issues inherent in traditional EMD. The most effective IMFs, those retaining over 30% of the original signal’s energy, were identified through correlation analysis, the percent fit procedure, and an energy-based damage index. These IMFs exhibited distinct amplitude and frequency shifts under damage conditions. This IMF selection strategy solves a critical ultrasonic SHM challenge: distinguishing true damage-induced energy loss from spurious nonlinear artifacts or noise. The proposed damage index advances this ability by systematically excluding negative DI values while quantifying only meaningful positive energy loss, thereby resolving a persistent diagnostic ambiguity in ultrasonic damage assessment.

Consistent degradation patterns were observed across time, frequency, and time-frequency domains, further validating the effectiveness of the method. Notably, as validated through energy-based damage indices, instantaneous amplitudes from the HSA proved more reliable than instantaneous frequency in damage identification. Additionally, frequency-domain analysis using the MHS and FFT revealed significant reductions in spectral magnitude within the 30–70 kHz range, corresponding to the transducer’s center frequency. While both methods successfully detected signal attenuation in damaged specimens, MHS provided superior resolution for capturing damage-related variations. Overall, the HHT framework demonstrated a reduction of up to 88% in signal component energy, confirming its advantage in nonstationary signal environments.

Supported by multi-domain statistical validations, the HHT framework demonstrates strong potential for early damage diagnosis in concrete. These findings highlight the feasibility of extending this approach to in-service concrete structures, where early damage detection is critical for ensuring safety and durability. Future work should focus on (1) optimizing IMF selection criteria, (2) extending the method to full-scale applications, and (3) refining energy-based damage metrics for practical field deployment. Additionally, integrating independent damage characterization techniques, such as micro-CT imaging, surface degradation scoring, or dynamic modulus testing, could further validate ultrasonic indices against physical deterioration. Exploring hybrid approaches that adapt DI methodologies for use in structures lacking baseline data, such as those incorporating surrogate signals, numerical simulations, statistical modeling, or machine learning, will also be critical. These efforts will enhance the reliability and applicability of CEEMDAN-HSA-based ultrasonic assessments for the non-destructive evaluation of concrete in real-world environments.

## Figures and Tables

**Table 1:** Comparison of signal processing techniques for ultrasonic NDT applications [29].

Method	Handles Non-Stationarity?	Handles Nonlinearity?	Adaptive?	Time-Frequency Resolution
FFT	No	No	No	Poor
Wavelet	Partial	Partial	Semi	Moderate
HHT	Yes	Yes	Yes	High

**Table 2:** Energy damage index values of the magnitude reduction in the MHS and fast Fourier transform (FFT) for the intact and damaged PMMA sample signals and their most effective IMFs, calculated in the frequency domain at the effective frequency ranges.

	MHS		FFT	
Signals	Effective Frequency Range	DI Value (%)	Effective Frequency Range	DI Value (%)
Original	30–70 kHz	74	30–60 kHz	68
IMF1	40–60 kHz	76	30–60 kHz	68
IMF2	20–60 kHz	78	40–60 kHz	71
IMF3	20–60 kHz	88	20–60 kHz	85

**Table 3:** Energy damage index values of the intact and damaged concrete sample signals and their most effective IMFs, calculated in the time domain at the effective time ranges.

Signals	DI Value (%)
Original	78
IMF3	78
IMF4	85
IMF5	87

**Table 4:** Energy damage index values of the instantaneous amplitude of the intact and damaged concrete sample signals and their most effective IMFs, calculated in the time–frequency domain at the effective time ranges.

Signals	Effective Time Range (ms)	DI Value (%)
Original	0.55–1.00	78
IMF3	0.55–0.70	77
IMF4	0.55–1.00	87
IMF5	0.50–1.50	87

**Table 5:** Energy damage index values of the magnitude reduction in the MHS and FFT for the intact and damaged concrete sample signals and their most effective IMFs, calculated in the frequency domain at the effective frequency ranges.

	MHS		FFT	
Signals	Effective Frequency Range	DI Value (%)	Effective Frequency Range	DI Value (%)
Original	30–70 kHz	82	30–80 kHz	72
IMF3	50–70 kHz	83	30–80 kHz	74
IMF4	40–70 kHz	78	40–80 kHz	72
IMF5	30–70 kHz	88	20–60 kHz	87

## Data Availability

The original contributions presented in this study are included in the article. Further inquiries can be directed to the corresponding author.
